# Inhibitory Potential of α-Amylase, α-Glucosidase, and Pancreatic Lipase by a Formulation of Five Plant Extracts: TOTUM-63

**DOI:** 10.3390/ijms24043652

**Published:** 2023-02-11

**Authors:** Quentin Haguet, Florian Le Joubioux, Vivien Chavanelle, Hugo Groult, Nathan Schoonjans, Cédric Langhi, Arnaud Michaux, Yolanda F. Otero, Nathalie Boisseau, Sébastien L. Peltier, Pascal Sirvent, Thierry Maugard

**Affiliations:** 1 UMR 7266 CNRS-ULR, LIENSs, Equipe BCBS, La Rochelle Université, Avenue Michel Crépeau, 17042 La Rochelle, France; 2 Valbiotis, R&D Center, 23 Avenue Albert Einstein, 17000 La Rochelle, France; 3 Valbiotis, R&D Center, 20-22 Rue Henri et Gilberte Goudier, 63200 Riom, France; 4 AME2P, STAPS, Université Clermont Auvergne, 5 Impasse Amélie Murat, 63001 Clermont-Ferrand, France

**Keywords:** plant extracts, type 2 diabetes, cardiometabolic diseases, enzyme assay, α-glucosidase

## Abstract

Controlling post-prandial hyperglycemia and hyperlipidemia, particularly by regulating the activity of digestive enzymes, allows managing type 2 diabetes and obesity. The aim of this study was to assess the effects of TOTUM-63, a formulation of five plant extracts (*Olea europaea* L., *Cynara scolymus* L., *Chrysanthellum indicum* subsp. *afroamericanum* B.L.Turner, *Vaccinium myrtillus* L., and *Piper nigrum* L.), on enzymes involved in carbohydrate and lipid absorption. First, in vitro inhibition assays were performed by targeting three enzymes: α-glucosidase, α-amylase, and lipase. Then, kinetic studies and binding affinity determinations by fluorescence spectrum changes and microscale thermophoresis were performed. The in vitro assays showed that TOTUM-63 inhibited all three digestive enzymes, particularly α-glucosidase (IC_50_ of 13.1 µg/mL). Mechanistic studies on α-glucosidase inhibition by TOTUM-63 and molecular interaction experiments indicated a mixed (full) inhibition mechanism, and higher affinity for α-glucosidase than acarbose, the reference α-glucosidase inhibitor. Lastly, in vivo data using leptin receptor-deficient (db/db) mice, a model of obesity and type 2 diabetes, indicated that TOTUM-63 might prevent the increase in fasting glycemia and glycated hemoglobin (HbA1c) levels over time, compared with the untreated group. These results show that TOTUM-63 is a promising new approach for type 2 diabetes management via α-glucosidase inhibition.

## 1. Introduction

Over the years, the worldwide prevalence of overweight and obesity has steadily increased, becoming a genuine pandemic. The growing incidence of patients with overweight/obesity (671 million people with obesity in 2016) represents an equal amount of potential patients with multiple metabolic pathologies in the future [1]. Currently, obesity is one of the leading causes of morbidity, including type 2 diabetes (T2D), prediabetes, cardiovascular diseases, and non-alcoholic fatty liver disease (NAFLD) [2,3,4,5]. Among them, T2D is a major concern due to its high prevalence (11.3% of the U.S. population in 2021) [6]. Hyperglycemia in T2D is caused by reduced insulin secretion and sensitivity, impaired glucose uptake, or increased glucose production associated with pathophysiological changes in multiple organs [7]. To manage these pathological states, various treatments are used with mixed results. Lifestyle changes lead to glycemia control improvement without side effects, but long-term adherence is generally low. On the other hand, drug-based treatments are not fully satisfactory, mainly due to their side effects and cost [8,9,10]. As the mechanisms underlying disorders such as components of metabolic syndrome (NAFLD, hypertension or T2D) are complex and interconnected, one often being a risk factor for developing another [11], multiple strategies are available to target these pathophysiological states, and it has now become common for people with T2D to be treated with multiple drug classes [12,13]. Among such strategies, we focused on reducing the intestinal absorption of carbohydrates and lipids to manage diabetes and obesity [14,15,16]. Carbohydrate absorption is regulated mainly by the digestive enzymatic activity, stomach emptying rate, alimentary bolus composition, and nutrient transporters [17]. It is largely accepted that inhibiting digestive enzymes helps to reduce post-prandial glycemia and to achieve long-term glycemia control. Therefore, their inhibition represents an attractive approach for T2D management [16]. Two classes of enzymes are involved in digestion: α-amylases (salivary and pancreatic) and α-glucosidases (intestinal maltase-glucoamylase and sucrase-isomaltase). They successively hydrolyze food polysaccharides into absorbable sugar units [18,19]. Similarly, although numerous parameters regulate lipid absorption, the reduction of gastric and pancreatic lipase activity is a therapeutic strategy that has already shown its efficacy in body weight management [15,20]. Three steps can be distinguished in the digestion of dietary lipids: emulsification, hydrolysis, and micellization. They are followed by lipid uptake by enterocytes [21]. A significant reduction in body weight in patients with obesity and diabetes strongly improves the control of glycemia [22]. Therefore, products capable of reducing both carbohydrate and lipid absorption could be used to effectively manage T2D. Plant extracts seem to be good candidates. Among the many plant biomolecules, phenolic compounds display very potent inhibitory effects on digestive enzymes [23]. In this context, TOTUM-63, a new patented tool, was developed to be used as a dietary supplement for management of obesity and T2D [24]. TOTUM-63 is a naturally biomolecule-rich formulation of five plant extracts: *Olea europaea* L. (olive tree), *Cynara scolymus* L. (artichoke), *Chrysanthellum indicum subsp. afroamericanum* B.L. Turner (chrysanthellum), *Vaccinium myrtillus* L. (bilberry), and *Piper nigrum* L. (black pepper). Several of the extracts included in this formulation are commonly used in nutraceutical formulations or dietary supplements [25,26,27,28], but the interest of this project is to test the specific combination of these five plant extracts. A first pilot clinical study in volunteers with overweight showed that TOTUM-63 has good safety and tolerability profiles and improves the glucose and insulin responses to a carbohydrate tolerance test [29]. The present study describes complementary experiments to bring insights into its mechanism of action. Specifically, we assessed TOTUM-63 effects in vitro on three enzymes involved in carbohydrate and lipid absorption (an α-glucosidase, an α-amylase, and a lipase) and in vivo on glucose homeostasis (db/db mice). In vitro experiments revealed that TOTUM-63 can significantly reduce the activity of the three digestive enzymes, particularly α-glucosidase, mainly through a mixed-type inhibition mechanism. The affinity of α-glucosidase for this formulation was higher than for acarbose, a commercial inhibitor. Furthermore, we demonstrated that many molecules and families of molecules present in TOTUM-63 might participate in its activity. Lastly, in vivo data in db/db mice showed that TOTUM-63 might prevent the rise in fasting glycemia and glycated hemoglobin levels.

## 2. Results

### 2.1. In Vitro Enzymatic Inhibitions

**Figure 1:**
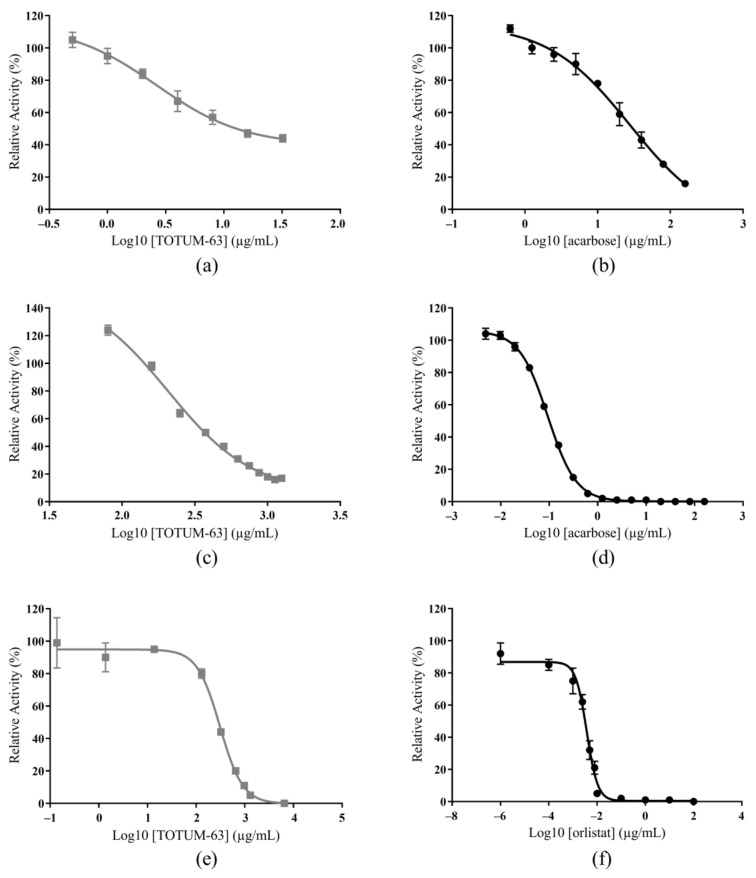
Inhibition of an α-glucosidase by TOTUM-63 (**a**) and acarbose (**b**) of an α-amylase by TOTUM-63 (**c**) and acarbose (**d**), and of a lipase by TOTUM-63 (**e**) and orlistat (**f**). Values are the mean ± SEM (the error bars represent the SEM with *n* = 2–9 and the SEM values were <10% of the mean values for acarbose or orlistat and <16% of the mean values for TOTUM-63).

The in vitro inhibitory effects of TOTUM-63 and of reference drugs (acarbose and orlistat, an α-glucosidase/α-amylase inhibitor and a lipase inhibitor, respectively) on α-glucosidase, α-amylase, and lipase activities are presented in Figure 1 and the IC_50_ values derived from these curves are in Table 1.

TOTUM-63 inhibited all three targets but displayed the highest effect on α-glucosidase (TOTUM-63 IC_50_ = 13.1 µg/mL and acarbose IC_50_ = 28.8 µg/mL). The inhibitory effect of TOTUM-63 on α-amylase was also good (IC_50_ = 380 µg/mL), although the acarbose effect was much stronger (IC_50_ = 0.110 µg/mL). TOTUM-63 inhibition of the lipase was in the same order of magnitude (IC_50_ = 276.5 µg/mL), but orlistat, the reference inhibitor, was much more effective (IC_50_ = 0.00345 µg/mL).

Due to the significant α-glucosidase inhibition by TOTUM-63, we then tested in vitro α-glucosidase inhibition by various compounds representative of the families of molecules identified in TOTUM-63 (see TOTUM-63 characterization Table 2) using the same methodology.

**Figure 2:**
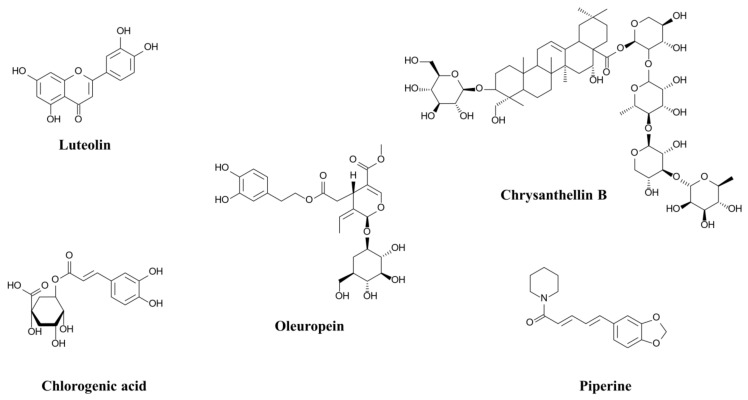
Chemical structures of the compounds tested for α-glucosidase inhibition.

Table 3 lists the IC_50_ values obtained for five compounds: chlorogenic acid, luteolin, oleuropein, chrysanthellin B, and piperine (Figure 2). These five compounds are representative of the major families of compounds identified in TOTUM-63.

The 5 tested compounds significantly inhibited α-glucosidase activity, as indicated by their IC_50_ values that ranged from 0.3 µg/mL for chlorogenic acid to 153.5 µg/mL for chrysanthellin B. Moreover, the inhibitory activity of 4 of these compounds (chlorogenic acid, luteolin, oleuropein, and piperine) was higher than that of acarbose (IC_50_ = 0.3 µg/mL, 0.4 µg/mL, 3.1 µg/mL, and 0.9 µg/mL, respectively, versus IC_50_ = 28.8 µg/mL for acarbose).

### 2.2. Kinetics and Mechanisms of α-Glucosidase Inhibition

**Figure 3:**
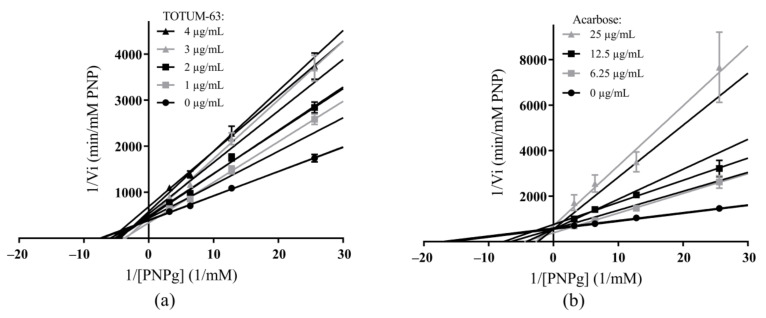
Lineweaver–Burk plots of α-glucosidase inhibition in the presence of TOTUM-63 (**a**) and acarbose (**b**). Values are the mean ± SEM (the error bars represent the SEM with *n* = 9–25 for all points in both groups and SEM values were ≤20% of the mean values for acarbose and <10% of the mean values for TOTUM-63). PNP, *p*-nitrophenol; PNPg, *p*-nitrophenyl-α-D-glucopyranoside.

Figure 3 shows the Lineweaver–Burk plots of α-glucosidase inhibition kinetics in the presence of various concentrations of substrate (*p*-nitrophenyl-α-D-glucopyranoside, PNPg) and of increasing concentrations of TOTUM-63 or acarbose.

Analysis of these data with the SigmaPlot software 12 (Systat Software Inc., San Jose, CA, USA) indicated a mixed inhibitory mechanism for TOTUM-63 and a competitive inhibitory mechanism for acarbose (as the main mechanisms, they obtained the best R² and Akaike Information Criterion corrected for small sample size, AICc values). As described for these mechanisms, the lines intersected to the left of the Y-axis and on the negative side of the X-axis for TOTUM-63, whereas they intersected on the Y-axis (X = 0) for acarbose. Moreover, with increasing TOTUM-63 concentrations, both the Y-intercept (1/Vmax) and slope (Km/Vmax) of the lines increased (decrease in velocity and substrate affinity), whereas with increasing acarbose concentrations, only the slope (Km/Vmax) of the lines increased (decrease in substrate affinity). Table 4 shows the main kinetic parameters obtained.

Lastly, the Ki value was lower for TOTUM-63 than for acarbose, and the factor α value for TOTUM-63 was 2.9, indicating better affinity for the free enzyme than for the enzyme–substrate complex.

### 2.3. Interactions with α-Glucosidase Using Fluorescence Spectrum Changes

To assess the products’ interactions with the enzyme, we recorded α-glucosidase fluorescence emission in the ultraviolet range (320–440 nm), in the absence and presence of TOTUM-63 or acarbose, upon excitation at 285 nm.

**Figure 4:**
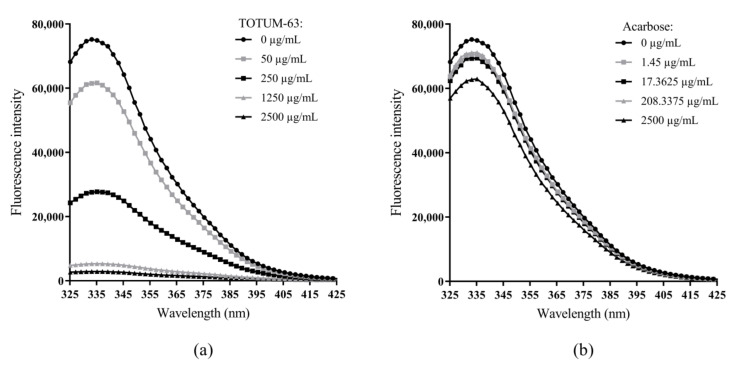
Fluorescence spectra of α-glucosidase in the presence of TOTUM-63 (**a**) and acarbose (**b**) at various concentrations, upon excitation at 285 nm and emission spectrum between 320 and 440 nm.

The fluorescence intensity gradually decreased with increasing concentrations of TOTUM-63 (Figure 4a). We observed a similar trend with acarbose, but with a less obvious effect, and the fluorescence intensity remained high even at the highest concentrations (Figure 4b). We did not observe any significant shift of the maximum emission wavelength of α-glucosidase in the presence of TOTUM-63 or acarbose.

### 2.4. Binding Affinity for α-Glucosidase Using Microscale Thermophoresis (MST)

As detailed by Jerabek-Willemsen et al., 2014 [30], MST is a technique to quantify biomolecular interactions based on thermophoresis. Thermophoresis is the directed movement of molecules through a temperature gradient. This phenomenon can be impacted by several molecular properties such as size, charge, hydration shell, or conformation of the molecule studied. Thus, this technique is very sensitive to any change in molecular properties and allows precise quantification of molecular events independent of the size or nature of the investigated sample. Therefore, MST can detect events such as the binding of molecules to enzymes. The experiment consisted in assessing changes in fluorescence signals caused by a laser that locally increased the temperature. The dissociation constant between a protein and a hypothetical ligand can be determined if an interaction occurs, as the temperature-induced behavior of the protein–ligand couple would differ from that of each free entity.

**Figure 5:**
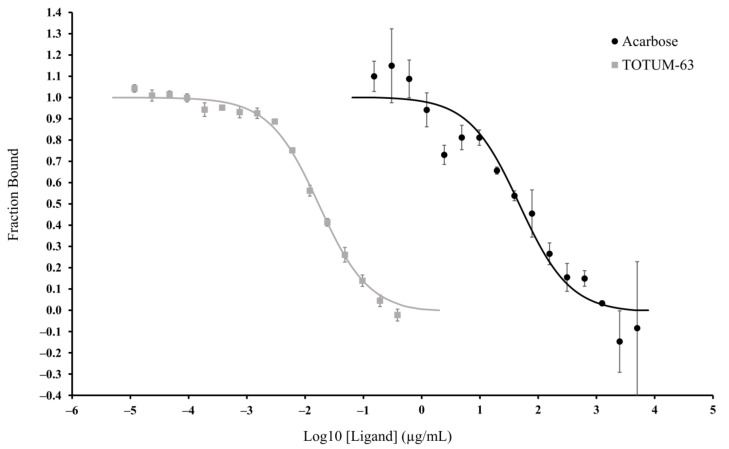
Fraction of TOTUM-63 and acarbose bound to α-glucosidase, measured by MST. Values are the mean ± SEM. *n* = 2 or 3 for all points in both groups. Raw fluorescence (in counts) for TOTUM-63 and variation of the normalized fluorescence (∆FNorm, in ‰) for acarbose were normalized by expressing them as the fraction bound to the target.

The binding affinity of TOTUM-63 and acarbose for α-glucosidase was assessed using MST. We analyzed the results of the experiments with TOTUM-63 (i.e., the ligand) using the Initial Fluorescence Analysis mode in the MO Affinity Analysis software (NanoTemper Technologies, Munich, Germany) because ligand binding directly led to a change in the initial fluorescence (ligand-induced fluorescence changes). We verified that the fluorescence changes recorded for α-glucosidase tagged with the fluorescent RED-NHS dye were ligand specific (TOTUM-63) and not due to any material loss. With acarbose we did not observe any change in the initial fluorescence and, therefore, we analyzed the data using the traditional MST mode based on the variation of the MST trace responses. Figure 5 shows the dose–response curves for TOTUM-63 and acarbose. The software analysis gave Kd values of 0.0173 µg/mL for TOTUM-63 and of 47.71 µg/mL for acarbose (i.e., 2758 times lower affinity for acarbose).

### 2.5. Glucose Homeostasis, Body Weight, and Body Composition in Diabetic db/db Mice

Body weight gain rate was significantly lower in db/db mice treated with TOTUM-63 (*n* = 10; 2.7% of TOTUM-63 added in the food for 6 weeks) than in the untreated control group (*n* = 10). Body weight started to be significantly lower in treated animals from week 4. The difference became more important at week 5 and week 6 (final measurement: 44.66 ± 1.99 g in the control group vs. 36.29 ± 1.66 g in the TOTUM-63 group, *p* < 0.0001). The differences in body weight were only due to fat mass reduction by 26.8% at week 6 (26.08 ± 1.52 g in the control group vs. 19.09 ± 1.13 g in the TOTUM-63 group, *p* < 0.01). Indeed, food intake remained similar between groups, and the lean mass was not significatively different at the end of the study (week 6) (17.02 ± 0.52 g in the control group vs. 16.47 ± 0.65 g in the TOTUM-63 group).

**Figure 6:**
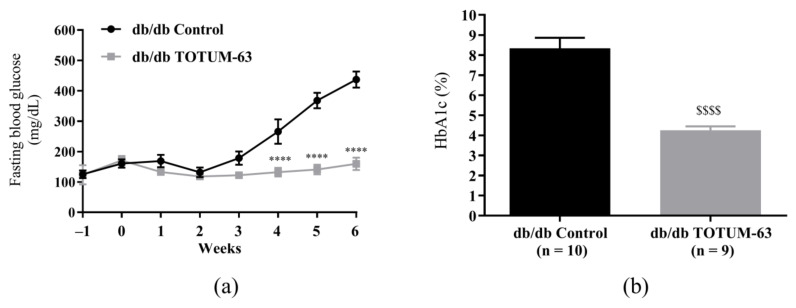
Evolution of fasting blood glucose levels (**a**) and HbA1c levels at week 6 (**b**) in db/db mice. **** *p* < 0.0001 (two-way ANOVA followed by the Sidak multiple comparisons test). $$$$ *p* < 0.0001 (Welch corrected unpaired *t*-test). Values are the mean ± SEM. *n* = 9 or 10 for all measurements. db/db, leptin receptor-deficient mice; HbA1c, glycated hemoglobin.

As observed for body weight, fasting glycemia was significantly lower in the TOTUM-63 group from week 4 compared with controls (132.1 ± 14.4 vs. 266.0 ± 40.0 mg/dL, *p* < 0.0001). At the study end (week 6), fasting blood glucose level was 2.74 times lower in the TOTUM-63 group (159.8 ± 20.4 mg/dL versus 437.4 ± 26.4 mg/dL in the control group, *p* < 0.0001) (Figure 6a). Glycohemoglobin (HbA1c) levels at week 6 were also significantly lower in the TOTUM-63 group than in controls (4.25 ± 0.20% vs. 8.33 ± 0.53%; −1.96%, *p* < 0.0001) (Figure 6b).

## 3. Discussion

In the present study, we showed that TOTUM-63 can inhibit some digestive enzymes related to the carbohydrate and lipid metabolisms. We observed the strongest effects of TOTUM-63 on α-glucosidase activity. We thoroughly explored this inhibitory activity through in vitro interaction and affinity studies. Then, in vivo experiments confirmed the potential of TOTUM-63 to prevent body weight gain and glycemia increase in a mouse model of obesity and T2D.

Acarbose is one of the most widely known and used T2D management drugs, particularly through the inhibition of digestive enzymes [31]. In vitro, acarbose strongly inhibited α-glucosidase (IC_50_ = 28.8 µg/mL), but the inhibitory effect of TOTUM-63 was stronger (IC_50_ = 13.1 µg/mL). The results obtained with acarbose are consistent with a previous study that reported comparable IC_50_ values [32]. As described in Table 2, the mixture of plant extracts in TOTUM-63 formulation is rich in phenolic compounds, terpenoids, and alkaloids. Any of these components may participate in the observed activities, as well as other non-listed constituents. Moreover, a synergy between components may exist [33,34]. Indeed, synergistic interactions are documented for biomolecules within a single plant extract, as well as between different plant extracts in a formulation. In these cases, the synergistic effects have been demonstrated by a better efficacy of the formulation than additional effects of the isolated constituents (biomolecules or plant extracts) [35]. Thus, we tested the inhibitory effect on α-glucosidase of five compounds that are representative of the families of molecules identified in TOTUM-63. The three phenolic compounds (chlorogenic acid, luteolin, and oleuropein), a terpenoid (chrysanthellin B: a saponin based on oleanolic acid) and an alkaloid (piperine) showed significant inhibitory activity (Table 3) that was stronger than that of acarbose for four of them (chlorogenic acid, luteolin, oleuropein, and piperine). Several studies have already evaluated the effects of chlorogenic acid and luteolin on α-glucosidase showing that these compounds have higher inhibitory activity than acarbose, the reference inhibitor [36,37,38]. The inhibitory activities of oleuropein and piperine on α-glucosidase have been less studied and the results are conflicting. Hadrich et al. [39] reported that oleuropein IC_50_ was two times higher than that of acarbose, while Dekdouk et al. [40] found the opposite. In our in vitro inhibition experiments, the IC_50_ of oleuropein was 10 times lower than that of acarbose. Similarly, Kumar et al. [41] showed that the IC_50_ of piperine was 28 times higher than that of acarbose. Conversely, Tolmie et al. [42] found similar inhibitory activities (Ki) for piperine and acarbose, and our in vitro data gave an IC_50_ for piperine 30 times lower than that for acarbose. Despite these differences, all studies showed a significant α-glucosidase inhibition by oleuropein and piperine. The inhibitory effect of chrysanthellin B on α-glucosidase was five times lower than that of acarbose but was still significant. We did not find any previous studies on α-glucosidase inhibition by chrysanthellin B. However, the effects of other molecules from the same sub-families (saponins based on oleanolic acid or oleanane) on this enzyme have been studied and the reported IC_50_ values were close to those of acarbose [43,44]. In addition, chlorogenic acid, luteolin, oleuropein, chrysanthellin B, and piperine are precursors or derivatives of several other compounds identified in TOTUM-63 (see Table 2) that could also potentially contribute to α-glucosidase inhibition. For instance, cyanidin (one of TOTUM-63 anthocyanins), apigenin, luteolin-7-O-glucoside, and oleanolic acid exert strong inhibitory effects on α-glucosidase [45,46,47,48]. Oleanolic acid showed the strongest effect with 162 times higher inhibitory activities on α-glucosidase compared with acarbose (IC_50_ = 4.09 µM for oleanolic acid and IC_50_ = 665.56 µM for acarbose) [49]. Moreover, other studies have demonstrated major or minor inhibitory effects on α-glucosidase for several other compounds identified in TOTUM-63: apigenin-7-O-glucuronide, dicaffeoylquinic acids (including cynarin), eriodictyol, marein, flavanomarein, and apigenin-6,8-C-diglucoside (vicenin 2) [50,51,52,53,54]. All these results confirm that α-glucosidase inhibition by TOTUM-63 is not due to a single specific molecule, but that many molecules and families of molecules present in the TOTUM-63 formulation contribute to this effect.

TOTUM-63 inhibitory effects on α-amylase and lipase are comparable with those reported by other authors who tested other plant extracts, but they were much lower than those observed with the reference drugs acarbose and orlistat [55,56,57]. However, it should be noted that excessive inhibition of α-amylase and lipase is associated with side effects. Therefore, the mild effect of TOTUM-63 on these enzymes could represent an advantage and might limit the risk of gastrointestinal disturbances and vitamin malabsorption, in particular [22,58,59]. Furthermore, as previously described for α-glucosidase, several studies have demonstrated significant inhibitory effects on α-amylase or lipase for many compounds identified in TOTUM-63. Significant α-amylase inhibitory activities have been shown for apigenin, apigenin-7-O-glucuronide, oleanolic acid, anthocyanins, monocaffeoylquinic acids (including chlorogenic acid), dicaffeoylquinic acids (including cynarin), luteolin, eriodictyol, and marein [49,50,51,52,55,60,61,62]. The family of compounds showing the strongest effect were the di-caffeoylquinic acids with 4.8, 6.2, 7.2, 9.4, and 11.4 times higher inhibitory activities on α-amylase than acarbose for cynarin, 1,5-dicaffeoylquinic acid, 3,4-dicaffeoylquinic acid, 3,5-dicaffeoylquinic acid, and 4,5-dicaffeoylquinic acid, respectively (based on IC_50_ values) [51]. Similarly, significant lipase inhibitory activities have been shown for apigenin, oleanolic acid, anthocyanins, monocaffeoylquinic acids (including chlorogenic acid), dicaffeoylquinic acids (including cynarin), luteolin-7-O-glucoside, luteolin, and Eriodictyol [63,64,65,66,67,68]. The highest lipase inhibitions were obtained using luteolin-7-O-glucoside and eriodictyol, two flavonoids, with more than two times higher inhibitory activities than orlistat for both (based on IC_50_ values) [68]. All these results confirm that α-amylase and lipase inhibitions by TOTUM-63 are not due to a single specific molecule, but that many molecules and families of molecules present in TOTUM-63 formulation contribute to these effects.

Then, we studied TOTUM-63 mechanism of action by monitoring its interaction with α-glucosidase because it was the most sensitive target enzyme. Analysis of α-glucosidase inhibition kinetics showed the expected results for acarbose, confirming its widely described competitive inhibitory mechanism [45]. The results obtained for TOTUM-63 fitted with a mixed inhibitory mechanism. However, given the various biomolecules contained in this formulation, many other inhibitory mechanisms may also be involved to a lesser extent [46,47,48,55,56,57,69]. Indeed, the mixed model could be partially explained by the activities of cyanidin, luteolin-7-O-glucoside, oleanolic acid, or apigenin, known to inhibit α-glucosidase according to this model [45,46,47,48]. For the other molecules with demonstrated inhibitory effect on α-glucosidase, various inhibition mechanisms have been described in the literature. Chlorogenic acid, luteolin, and piperine are considered mixed, non-competitive, and mixed inhibitors, respectively [36,38,42]. Furthermore, molecular docking simulation studies showed that apigenin, luteolin, and cyanidin bind to sites close to the active site of α-glucosidase, which may induce conformational changes and/or a channel closure to prevent the substrate access, eventually leading to α-glucosidase inhibition [38,45,47]. In the same way, Deng et al. [49] performed docking simulation for α-glucosidase using oleanolic acid oxime ester derivatives as potential inhibitors and they demonstrated the oleanolic acid part of the derivative located inside of the enzyme active site. Regarding caffeoylquinic acids (mono- or di-), molecular docking results showed different binding energies and modes with α-glucosidase depending on their structures and especially the caffeoyl groups’ distribution [70]. Thus, the various molecules identified in TOTUM-63 would act on several sites of the enzymes. This seems to confirm that the mechanism of α-glucosidase inhibition by TOTUM-63 is influenced by all the compounds present in TOTUM-63, which predominantly exhibit mixed inhibition mechanisms.

The study of kinetic parameters revealed that the affinity of TOTUM-63 inhibitory biomolecules (Ki) for α-glucosidase was 1.67 times higher than that of acarbose, in agreement with the IC_50_ values (~2.2 times lower). The MST technique revealed that both TOTUM-63 and acarbose could bind to α-glucosidase, modifying the enzyme response to a local temperature gradient. However, TOTUM-63’s affinity for α-glucosidase was much higher than that of acarbose (by 2758 times). Two hypotheses could be made. The main active TOTUM-63 component (for this target) has a very high capacity to bind to α-glucosidase, but a relatively low effect on its activity compared with acarbose, thus leading to ~two times higher inhibitory effect (IC_50_ and Ki). Alternatively, many molecules in the plant extract mixture could bind to α-glucosidase, possibly at multiple sites; however, most of them do not exert any effect on the enzyme activity. Indeed, changes in an enzyme’s endogenous fluorescence intensity and spectra can be caused by quenching due to binding followed by conformational changes, or by a change in the enzyme microenvironment, such as a small distance (<10 nm) between the ligand and the enzyme fluorescent amino acids [71,72]. Similarly, for the MST results, the more important fluorescence spectra changes upon excitation at 280 nm in the presence of TOTUM-63, compared with acarbose, could be explained by multiple molecules interacting with α-glucosidase elsewhere than at the active site. This would be in line with the allosteric regulation expected for the mixed inhibition shown by TOTUM-63. Conversely, acarbose can only bind to the active site on α-glucosidase, thus causing fewer fluorescence changes [46,73,74].

Based on these results, we hypothesized that potent activities could also be observed in vivo. Therefore, we assessed TOTUM-63’s effects on glucose homeostasis and on body weight in db/db mice, a model of T2D [75]. At the end of the study, fasting blood glucose and HbA1c levels were significantly lower in mice supplemented with TOTUM-63 for 6 weeks compared with the control group, suggesting an improvement of glucose homeostasis. Moreover, we observed reduced fat mass gain, resulting in lower body weight gain in supplemented animals. Interestingly, these beneficial effects are often reported when studying the effects of drugs capable of inhibiting digestive enzymes such as glucosidases or lipases in vivo. Specifically, α-glucosidase inhibitors have shown beneficial effects in blunting post-prandial hyperglycemia, resulting in modest improvements in HbA1c in db/db mice [76]. This class of drugs works by reducing the activity of sucrase and maltase in the small intestine [77], resulting in less monosaccharides being produced out of complex carbohydrates. Polysaccharides are unable to be up-taken by the intestinal transporters, hence α-glucosidase inhibitors ingestion results in less sugar being absorbed overall. Interestingly in humans, long-term HbA1c reduction is also reported [78], sometimes associated to a modest decrease in body weight as well [79]. Similarly, pharmacologic inhibition of pancreatic lipase is a common and approved strategy to reverse obesity, resulting in body weight loss in humans [80]. Here again, the mechanism involved in the anti-obesity effects is rather simple, as lipase inhibitors prevent part of the ingested triglycerides to be hydrolyzed into simple monoacylglycerol and fatty acids, the only forms able to be transported by the enterocyte [21]. This results in more fat being excreted into the feces, and hence, less energy being absorbed. Interestingly, just like in the present study, this reduction in body weight is associated with improved fasting glycemia and reduced HbA1c levels in humans, suggesting an improvement of glucose control [80].

Furthermore, in addition to all the hypotheses related to the digestive enzymes previously described, there are several other hypotheses that may explain the effect on the HbA1c level of TOTUM-63, a polyphenol-rich formulation. One of these hypotheses could be a direct effect of the phenolic compounds in TOTUM-63 on the glycation of hemoglobin. Indeed, several studies have already demonstrated the deglycation effects of several flavonoids such as luteolin on different proteins including hemoglobin [81,82].

The pathophysiology of metabolic diseases such as T2D being complex, interconnected, and rooted in several key organs, this work does not allow discrimination of the individual in vitro effect on each enzyme on glucose control or fat mass. Incidentally, a reduction in adiposity alone is a strong stimulus for improvement of glucose control [83], which has subsequently made body weight management an integral part of the recommendations of major health organizations worldwide for people with prediabetes or T2D, to improve glucose metabolism [84]. Hence, even though we could not demonstrate a direct and causal link between in vitro and in vivo effects, the fact that the inhibitory effect on digestive enzymes translates into improvements of body composition and glucose homeostasis in a well-known and extensively used mouse model of T2D certainly adds to the understanding of the complex mechanisms involved in the effects of Totum-63 in vivo.

## 4. Materials and Methods

### 4.1. Materials and Reagents

α-Glucosidase (maltase from *Saccharomyces cerevisiae*), α-amylase (type VI-B, from porcine pancreas), lipase (type II, from porcine pancreas), *p*-nitrophenyl-α-D-glucopyranoside (PNPg), *p*-nitrophenol (PNP), 2-chloro-4-nitrophenyl-4-O-b-D-galactopyranosyl-a-D-maltoside (GalG_2_CNP), 2-chloro-4-nitrophenol (CNP), 4-methylumbelliferyl oleate (4-MUO), 4-methylumbelliferone (4-MU), orlistat, acarbose, pluronic F-127, Folin–Ciocalteu reagent, Na_2_CO_3_, Ca(OAc)_2_, NaCl, gallic acid, potassium phosphate buffer, phosphate-citrate buffer, chlorogenic acid, luteolin, oleuropein, piperine, absolute ethanol, and dimethyl sulfoxide (DMSO) were obtained from Merck/Sigma-Aldrich (Darmstadt, Germany). Chrysanthellin B was purchased from Extrasynthese (Genay, France). 2-(N-morpholino)-ethanesulfonic acid (MES) buffer was purchased from ThermoFisher Scientific (Waltham, Massachusetts, USA). NaN3 was obtained from Carlo Erba Reagents (Val-de-Reuil, France). Pure water was obtained using an Evoqua ultra-pure water production system (Water Technologies, Günzburg, Germany). The RED-NHS protein labeling kit was from NanoTemper Technologies (Munich, Germany).

TOTUM-63 (Batch No. V190033) was supplied as a powder by VALBIOTIS (Perigny, France). This product is formulated with five standardized plant extracts (*Olea europaea* L., *Cynara scolymus* L., *Chrysanthellum indicum subsp. afroamericanum* B.L.Turner, *Vaccinium myrtillus* L., and *Piper nigrum* L.). Voucher specimens of batches from TOTUM-63 and all plant extracts were deposited and stored in the VALBIOTIS sample library. Table 2 shows the phytochemical characterization of TOTUM-63. Total phenolic compound levels (in gallic acid equivalent) were assessed using the Folin–Ciocalteu colorimetric method [85] and a more precise characterization of phytochemical compounds was performed by HPLC-UV/Visible-MS using a 1200 LC system with a 6110 Single Quad MS-ESI detector (Agilent Technologies, Santa Clara, CA, USA) with a C18 Prodigy reversed-phase column (250 mm × 4.6 mm, 5 μm; Phenomenex, USA).

### 4.2. In Vitro Enzymatic Inhibition Assays

#### 4.2.1. α-Glucosidase Inhibition Assay

α-Glucosidase hydrolytic activity was assessed by monitoring the conversion of *p*-nitrophenyl-α-D-glucopyranoside (PNPg) into *p*-nitrophenol (PNP). PNP levels were quantified at 405 nm. A commercial reference inhibitor, acarbose, was used as positive control, and potassium phosphate buffer (0.1 M, pH 6.8) was used as a reaction buffer. A series of PNP dilutions in buffer was used to generate the standard curve to convert PNP absorbance values into concentrations. The final α-glucosidase concentration corresponded to an activity of 30 µM of PNP/min for negative control without inhibitor. In each microplate well, 20 µL of test products diluted in a 50:50 (v:v) water:ethanol mixture (or 20 µL of this solvent mixture for the negative controls), 20 µL of α-glucosidase in buffer (or 20 µL of buffer for the blanks), and 100 µL of buffer were incubated in a microplate reader at 37 °C for 10 min. Then, 20 µL of PNPg substrate (2.5 mM in buffer) was added to each well to initiate the reaction. A FLUO Star Omega (BMG LabTech, Champigny sur Marne, France) 96-well microplate reader, thermostatically controlled at 37 °C, was used to measure the absorbance at 405 nm, every minute for 30 min. The relative enzymatic activity was calculated using Equation (1), where the negative control activity was the initial reaction rate in the absence of any compound, and the residual activity was the initial reaction rate in the presence of test products. The IC_50_ of each compound corresponded to the lowest concentration at which the glucosidase activity was halved.

(1) $$\%\;\mathrm{Relative}\;\mathrm{activity}=\frac{\mathrm{Residual}\;\mathrm{activity}}{\mathrm{Negative}\;\mathrm{control}\;\mathrm{activity}}\times 100\;$$

#### 4.2.2. α-Amylase Inhibition Assay

α-amylase hydrolytic activity was assessed by monitoring the conversion of 2-chloro-4-nitrophenyl-4-O-b-D-galactopyranosyl-a-D-maltoside (GalG_2_CNP) into 2-chloro-4-nitrophenol (CNP). CNP levels were quantified at 400 nm. A commercial reference inhibitor, acarbose, was used as the positive control, and MES buffer (50 mM, pH 6), with 5 mM Ca(OAc)_2_, 51.5 mM NaCl, and 152 mM NaN_3_, was used as the reaction buffer. A series of CNP dilutions in buffer was used to generate the standard curve to convert absorbance values into CNP concentrations. The final α-amylase concentration corresponded to an activity of 200 µM CNP/min for the negative control without inhibitor. In each microplate well, 20 µL of test products diluted in a 50:50 (v:v) water:ethanol mixture (or 20 µL of this solvent mixture for the negative controls), 20 µL of α-amylase in buffer (or 20 µL of buffer for the blanks), and 100 µL of buffer were incubated in a microplate reader at 37 °C for 10 min. Then, 20 µL of GalG_2_CNP substrate (0.3125 mM in buffer) was added to each well to initiate the reaction. A FLUO Star Omega (BMG LabTech, Champigny sur Marne, France) 96-well microplate reader, thermostatically controlled at 37 °C, was used to measure the absorbance at 400 nm, every minute for 30 min. The relative enzymatic activity was calculated using Equation (1). The IC_50_ of each compound corresponded to the lowest concentration at which the amylase activity was halved.

#### 4.2.3. Lipase Inhibition Assay

Lipase hydrolytic activity was assessed by monitoring the conversion of 4-methylumbelliferyl oleate (4-MUO) into 4-methylumbelliferone (4-MU). The enzymatic activity was determined by measuring the 4-MU fluorescence signal at 460 nm (after excitation at 355 nm) over time. A commercial reference inhibitor, orlistat, was used as the positive control, and citrate-phosphate buffer (0.1 M, pH 7.4) was used as the reaction buffer. A series of 4-MU dilutions in buffer was used to generate the standard curve to convert absorbance values into 4-MU concentrations. A 4 g/L solution of lipase in buffer was centrifuged at 5000× *g* and 10 °C for 10 min, then the supernatant was aliquoted. The final on-well concentration used in the assay was 250 mg/L. In each microplate well, 20 µL of test products diluted in a 10:90 (v:v) DMSO:water mixture (or 20 µL of this solvent mixture for the controls), 20 µL of lipase in buffer (or 20 µL of buffer for the blanks), and 110 µL of buffer were incubated at 37 °C in a microplate reader for 10 min. Then, 10 µL of 4-MUO substrate (48 µM in DMSO) was added to each well to initiate the reaction. Fluorescence was measured at 355 nm/460 nm (excitation/emission) using a FLUO Star Omega (BMG LabTech, Champigny sur Marne, France) 96-well microplate reader, thermostatically controlled at 37 °C, every 30 s for 15 min. The relative enzymatic activity was calculated using Equation (1). The IC_50_ of each compound corresponded to the lowest concentration at which the lipase activity was halved.

### 4.3. α-Glucosidase Inhibition Kinetics

α-glucosidase inhibition kinetics was investigated in vitro using the method described in Section 4.2.1. Inhibition models were determined by analysis of the Lineweaver–Burk plots using four concentrations of substrate and four concentrations of test products (including a “0”, without inhibitor). All initial rates were fitted to all enzymatic kinetic models with one substrate and one inhibitor provided by the Enzyme Kinetics Module of Sigma Plot 12 (Systat Software Inc., San Jose, CA, USA), using non-linear least-square regression models to determine the inhibition model and calculate the inhibitory constant (Ki). The mechanism of α-glucosidase inhibition by TOTUM-63 was confirmed using Lineweaver–Burk plots (1/[S] against 1/v). The acarbose inhibition model was verified and compared with the literature to validate the obtained results [86].

Equations (2) and (3) (below) were used for data analysis and corresponded to the inhibition models that best fitted our data according to the SigmaPlot software 12 (Systat Software Inc., San Jose, CA, USA) [73]. Equation (2), for acarbose, corresponded to the competitive (full) inhibition mechanism where v corresponds to the reaction velocity (also called reaction rate), Vmax to the maximum reaction velocity, Km to the amount of substrate required to obtain half of the Vmax (also called Michaelis constant), Ki to the inhibitor dissociation constant, [S] to the substrate concentration, and [I] to the inhibitor concentration. Equation (3), for TOTUM-63, corresponded to the mixed (full) inhibition mechanism with a new factor, Ki’, introduced compared with Equation (2). Ki’ corresponds to the second inhibitor dissociation constant because, in a mixed model, the constants for E + I ↔ EI and ES + I ↔ ESI are different. Factor α, given by SigmaPlot (Systat Software Inc., San Jose, CA, USA), represents the equilibrium between Ki’ and Ki.

(2) $$v\;=\frac{\mathrm{Vmax}}{1+\left(\frac{\mathrm{Km}}{\left[S\right]}\right)\times \left(1+\frac{\left[I\right]}{\mathrm{Ki}}\right)}$$

(3) $$v\;=\frac{\mathrm{Vmax}}{\left(1+\frac{\left[I\right]}{{\mathrm{Ki}}^{’}}\right)+\frac{\mathrm{Km}}{\left[S\right]}\times \left(1+\frac{\left[I\right]}{\mathrm{Ki}}\right)}$$

### 4.4. Determination of Interactions with α-Glucosidase Based on Fluorescence Spectrum Changes

The fluorescence spectra of α-glucosidase in the absence and presence of TOTUM-63 or acarbose were recorded with a microplate fluorescence spectrophotometer (Spark 10M, Tecan, Männedorf, Switzerland). Potassium phosphate buffer (0.1 M, pH 6.8) was used as the reaction buffer. The final α-glucosidase concentration in the wells was 1.275 mg/mL. For each microplate well, 20 µL of test product in a 50:50 (v:v) water:ethanol mixture (or 20 µL of this solvent mixture for the controls without products), 100 µL of α-glucosidase in buffer (or 100 µL of buffer for the blanks), and 40 µL of buffer were incubated at 25 °C in a microplate reader for 5 min. Then, fluorescence was measured at an excitation of 285 nm and an emission scan ranging from 320 nm to 440 nm.

### 4.5. Determination of the Binding Affinity for α-Glucosidase Using Microscale Thermophoresis (MST)

The binding affinity of TOTUM-63 and acarbose for α-glucosidase was assessed using MST. α-glucosidase was labeled with the fluorescent RED-NHS dye using the RED-NHS protein labeling kit (NanoTemper Technologies, Munich, Germany) according to the manufacturer’s instructions. Briefly, the dye and enzyme were incubated and the tagged α-glucosidase was purified. The resultant tagged solution was then diluted in PBS supplemented with Pluronic F-127 to achieve a final α-glucosidase concentration of 100 nM with 0.01125% Pluronic F-127 in the capillaries. The 2 test products, TOTUM-63 and acarbose, were prepared at their respective optimized concentrations of 0.3125 g/L and 10 g/L before 2-fold serial dilutions for 15 times to obtain ligand concentrations ranging from 0.3125 g/L to 9.5367 µg/L and from 10 g/L to 305.1758 µg/L, respectively. Standard capillaries for red fluorescence emission recording were filled with a 1:1 (v/v) mixture of tagged enzyme and diluted ligand. Fluorescence was measured using the NanoTemper Monolith NT.115 instrument (NanoTemper Technologies, Munich, Germany) at 37 °C, with a fluorescence excitation power of 80% and MST power set to Medium. Data were analyzed with the NanoTemper analysis software (NanoTemper Technologies, Munich, Germany).

### 4.6. Animals and Diet

Five-week-old male db/db mice (BKS(D)-Leprdb/JOrlRj) were purchased from JANVIER LABS (Le Genest-Saint-Isle, France) and housed in individual cages with a normal 12 h/12 h light/dark cycle). After 1 week of acclimatization, 20 5-week-old male db/db mice (BKS(D)-Leprdb/JOrlRj) were randomly assigned to Control (*n* = 10) or TOTUM-63 (*n* = 10) groups to obtain 2 groups with comparable fasting glycemia and body weight. Animals belonging to the control group were fed a standard A03 diet enriched with 3% corn oil (Safe, France). Animals in the TOTUM-63 group were fed the same diet supplemented with 2.7% *w*/*w* TOTUM-63, incorporated directly into the chow by the manufacturer. The optimum dose (2.7%) was chosen during our preliminary experiments when plant extracts were tested individually and in combination at different ratios (unpublished data), to result in the final composition. All procedures were approved by the local ethics committee (C2EA-02, Auvergne, France) under number 00898.01.

### 4.7. Glucose Homeostasis, Body Weight, and Body Composition in Diabetic db/db Mice

Body weight and food intake were recorded weekly. After an overnight fast, glycemia was measured weekly using a drop of whole blood taken from the tail and an Accu-Chek Performa blood glucose meter (Roche, Mannheim, Germany). After 6 weeks of treatment, fat and lean mass were recorded by MRI (EchoMRI 3-in-1 instrument; Echo Medical Systems, Houston, TX, USA). Finally, animals were fasted overnight (16 h) and euthanized by cervical dislocation. Whole blood was harvested right after the death of the mice and a small portion was collected in an EDTA-treated tube and put on ice until analysis. Glycated hemoglobin (HbA1c) levels were measured in EDTA-treated whole blood with a mouse Hemoglobin A1c (HbA1c) Assay Kit (Crystal Chem, Elk Grove Village, IL, USA) by comparing absorbance at 700 nm in a spectrometer with Hba1c controls according to the instructions of the manufacturer.

### 4.8. Statistical Analysis of In Vivo Data

Prism V.7.0 and 8.0 (GraphPad Software, San Diego, CA, USA) and the SAS/STAT software V.9.4 (SAS Institute, Cary, NC, USA) were used for statistical analyses and figure drawing. For pair comparisons, the Shapiro–Wilk normality test was used to determine whether data followed a Gaussian distribution, and then the F-test (Fisher) was used to compare variances. If normal distribution was assumed and variances were homogenous, the unpaired t-test was used to compare groups. If variances were heterogenous, the unpaired t-test with Welch correction was used. If data were not consistent with a Gaussian distribution, the Mann–Whitney test was used. For measurements repeated over time, differences were tested using a repeated-measure two-way ANOVA followed by the Sidak post hoc test for multiple comparisons if the interaction was statistically significant. Values are presented as the mean ± SEM, unless specified otherwise. Differences were considered statistically significant at *p* < 0.05.

## 5. Conclusions

Controlling post-prandial hyperglycemia and hyperlipidemia allows managing T2D. Therefore, it is important to identify non-drug strategies to control these parameters. Our in vivo study in db/db mice (a model of obesity and T2D) showed that TOTUM-63, a naturally biomolecule-rich formulation of five plant extracts, improves glycemic control with fasting blood glucose and HbA1c levels were significantly lower compared with the control group. An inhibition of digestive enzyme activity may partly explain the improved glucose homeostasis. Indeed, in vitro study revealed that TOTUM-63 can reduce digestive enzyme activity, particularly α-glucosidase activity with a mixed-type inhibition mechanism. TOTUM-63 acted on targets regulated by commonly used drugs (e.g., acarbose), with higher activity than these drugs which bodes well for its potential use in humans [31]. Thus, TOTUM-63 is an attractive candidate for the management of hyperglycemia and T2D.

## Figures and Tables

**Table 1:** IC_50_ of TOTUM-63 and reference drugs for α-glucosidase, α-amylase, and lipase inhibition.

	IC_50_ Values in µg/mL		
Tested Products	α-Glucosidase	α-Amylase	Lipase
TOTUM-63	13.1	380.0	276.5
Acarbose	28.8	0.110	-
Orlistat	-	-	0.00345

**Table 2:** Phytochemical characterization of TOTUM-63.

Compound Types (Sorted by Families)	Extract Content (g/100 g)
Total phenolic compounds	9.1
Total anthocyanins	0.964
Monocaffeoylquinic acids	1.073
Chlorogenic acid	0.645
Other monocaffeoylquinic acids	0.428
Dicaffeoylquinic acids	0.917
Cynarine	0.112
Other dicaffeoylquinic acids	0.805
Caffeic acid	0.019
Oleuropein	3.645
Oleuropein isomers	0.519
Hydroxytyrosol	0.454
Luteolin	0.030
Luteolin-7-O-glucoside	0.656
Luteolin-7-O-glucuronide	0.440
Apigenin	0.016
Apigenin-7-O-glucoside	0.093
Apigenin-7-O-glucuronide	0.324
Apigenin-6-C-glucoside-8-C-arabinoside (Shaftoside)	0.029
Apigenin-6,8-C-diglucoside (Vicenin 2)	0.060
Eriodictyol	0.008
Eriodictyol-7-O-glucoside	0.590
Marein and Flavanomarein	0.318
Maritimein	0.129
Rutin	0.014
Verbascoside	0.046
Terpenes and terpenoids	
Oleanolic acid	2.004
Saponins	
Chrysanthellin A	0.553
Chrysanthellin B	0.507
Iridoids	
Oleoside	0.290
Alkaloids	
Piperine	0.007

**Table 3:** IC_50_ of five phytochemical compounds for α-glucosidase inhibition.

Tested Compounds	IC_50_ Values in µg/mL
Chlorogenic acid	0.3
Luteolin	0.4
Oleuropein	3.1
Chrysanthellin B	153.5
Piperine	0.9

**Table 4:** Kinetic parameters of α-glucosidase inhibition in the presence of TOTUM-63 or acarbose.

Parameters	TOTUM-63	Acarbose
Inhibition type	Mixed (full)	Competitive (full)
Vmax (mM/min)	0.00259	0.00182
Km (mM)	0.14	0.06
Ki (µg/mL)	2.7	4.5
Factor α	2.9	none

Vmax, maximum reaction velocity; Km, amount of substrate required to obtain half of the Vmax (also called Michaelis constant); Ki, inhibitor dissociation constant; Factor α, equilibrium between Ki’ (the second dissociation constant in the mixed model) and Ki.

## Data Availability

The data presented in this study are available on request to the corresponding author.
